# Reducing Physician Burnout Through Workflow Redesign: A Quality-Improvement Initiative

**DOI:** 10.7759/cureus.85799

**Published:** 2025-06-11

**Authors:** Jennifer Goebel, Jessica Sidle, Alma Aspiras, Leah Fow, Molly McCann-Pineo, Timmy Li

**Affiliations:** 1 Emergency Department, Northwell Health, New Hyde Park, USA; 2 Case Management, Northwell Health, New Hyde Park, USA; 3 Internal Medicine, Northwell Health, New Hyde Park, USA; 4 Statistics, Northwell Health, New Hyde Park, USA

**Keywords:** administrative burden, interdisciplinary collaboration, patient safety, physician well-being, quality improvement, workflow optimization, work-related burnout

## Abstract

Introduction

Staff well-being is a critical element in any healthcare organization’s framework. Improving provider and staff well-being is increasingly recognized as essential to high-quality care. Burnout among healthcare workers is often driven by excessive administrative demands, inefficient workflows, and time-consuming clerical tasks. These burdens take time away from meaningful patient interactions and clinical decision-making.

At Huntington Hospital, clerical workload, especially tasks related to electronic health records (EHRs) and manual documentation, was identified as a key source of stress. To better understand the contributors to burnout, the hospital administered the Maslach Burnout Inventory (MBI) to all hospitalists. The MBI evaluates emotional exhaustion, depersonalization, and reduced personal accomplishment, offering a comprehensive assessment of professional well-being.

Method

To address this issue, a multi-disciplinary wellness learning community was formed to identify and implement evidence-based strategies to support staff well-being. The hospitalist group unanimously chose to focus on improving the workflow for the 3122 form, a handwritten discharge document required for patients transitioning to assisted living facilities. This form included evaluations of Activities of Daily Living (ADLs), therapy needs, home care requirements, and a medication list-all of which had to be completed manually by physicians.

After multiple discussions with social work, case management, and peer institutions, it was decided that social workers would complete the sections of the form within their scope, such as ADLs and support needs. Medication lists were printed directly from the electronic medical record (EMR) and attached to the form to improve accuracy and efficiency. Hospitalists reviewed the completed form before it was submitted. This new workflow was piloted on one unit, with interest quickly spreading to other teams.

Results

With an 83% response rate to the MBI among hospitalists (33 out of 40 hospitalists), results revealed that workload was the primary driver of burnout. Following the workflow redesign, a two-question survey was conducted with hospitalists to assess the impact of the intervention. The survey asked physicians whether removing the 3122 form as a physician task had decreased their workload and whether they supported further collaborations with the Wellness Learning Community.

76% of respondents (n=21) agreed or strongly agreed that the change reduced their workload. An equal percentage supported ongoing efforts with the Wellness Learning Community to develop future process improvements and well-being initiatives.

Conclusion

This project highlights how small, targeted workflow changes can have a meaningful impact on clinician well-being. By reducing administrative burden and aligning task responsibility with appropriate team members, physicians were able to redirect time and energy toward patient care. The shift from handwritten documentation to EMR-generated materials also improved efficiency and reduced the risk of transcription errors.

Interdisciplinary collaboration played a key role in the project’s success. By engaging social work, case management, and frontline providers in the process, the hospital was able to design a more sustainable and effective solution. Focusing on staff well-being through operational redesign promotes a more resilient, satisfied, and safer healthcare workforce.

## Introduction

Staff well-being is a critical element in any organization’s framework, particularly in healthcare settings. The Quadruple Aim, a key healthcare delivery framework, emphasizes improving provider and staff well-being as one of its core pillars [1]. 

The 2022 U.S. Surgeon General’s advisory Addressing Health Worker Burnout underscored the need for a comprehensive societal approach to addressing burnout. It called for reducing administrative duties, focusing on human-centered health technology, and redesigning payment models to value provider-patient interactions. The advisory emphasized that providers currently spend two hours on clerical tasks for every hour spent with patients [2]. Similarly, the National Academy of Medicine highlighted the importance of streamlining workflows and minimizing manual tasks to allow physicians to engage in meaningful, focused care, often referred to as "deep work." Evidence suggests that re-engineering workflows and implementing team-based care models could reduce burnout, enhance provider well-being, and restore more time for direct patient care [2,3]. 

Clerical burdens, particularly those tied to electronic health records (EHRs), have been identified as the leading cause of burnout [4]. A study by DiGiorgio et al. revealed that residents spent up to 20 hours per on-call shift interacting with EHRs, with nine of those hours spent on clerical tasks instead of patient care [5]. This highlights the need for improving workflow efficiency and reducing administrative burdens to combat burnout and enhance physician well-being. 

To better understand the drivers of burnout among hospital staff, Huntington Hospital distributed the Maslach Burnout Inventory (MBI) to all its hospitalists. The MBI is one of the most widely used tools for measuring burnout in healthcare providers. It assesses three key dimensions of burnout: 1) emotional exhaustion, 2) depersonalization, and 3) reduced personal accomplishment. Emotional exhaustion refers to the feeling of being overextended and drained of emotional resources, while depersonalization involves a sense of cynicism or detachment from patients [6]. Reduced personal accomplishment refers to the feeling of incompetence and a lack of achievement in one’s work. The MBI provides a detailed snapshot of how healthcare professionals are coping with the emotional and psychological demands of their work [7].

## Materials and methods

A multi-disciplinary wellness learning community was established at our hospital to create evidence-based practices that best support staff well-being. Knowing that the number one driver of burnout was workload in our hospitalist group, the hospitalists unanimously wanted to tackle the workflow process of the 3122 form (Return to Assisted Living Medical Evaluation Form). Previously, hospitalists were required to complete this handwritten form for each patient discharged to an assisted living facility, taking them away from direct patient care. This form includes the patient’s diagnoses, handwritten medications requisition, allergies, mental status, Activities of Daily Living (ADLs), necessary therapies, and home care needs. The objective was to reduce this administrative burden, allowing hospitalists to focus more on their core responsibilities and patient interactions. Discussions were held with our social work and case management teams, as well as with similar groups at neighboring hospitals in the same system, to understand their workflows regarding this form. 

After several multi-disciplinary meetings, the decision was made to streamline the process by assigning responsibility for the form to the social work team. This was decided because the form included questions about ADLs and other areas that are within their scope of practice. Medications, previously handwritten by hospitalist staff, were printed from the electronic medical record (EMR) and attached to the form, aligning with practices at other hospitals and improving patient safety while reducing staff workload. By printing medication information directly from the EMR, the hospital also aimed to reduce transcription errors and improve the accuracy of patient data. Hospitalists would then review the form before it was sent to assisted living facilities. The revised workflow was piloted on one floor, and even before the pilot was complete, other hospitalists expressed interest in adopting the process on their own floors. 

## Results

The MBI was sent out to our hospitalist physicians at our large community hospital, and with a 83% response rate (33 out of 40 hospitalists), the results revealed that workload was the primary driver of burnout. 

**Figure 1:**
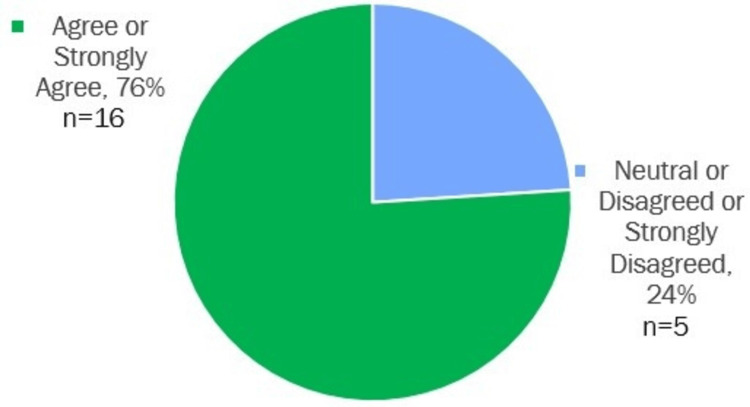
Removing the 3122 form as a physician task has decreased my workload burden

A two-question post-implementation survey was conducted with the hospitalist group, asking them to rate the following statements: "Removing the 3122 form as a physician task has decreased my workload burden" (Figure 1) and "I recommend partnering with the Wellness Learning Community for further process changes and program development." The results were overwhelmingly positive. 

76% respondents either agreed or strongly agreed that removing the 3122 form as a physician task reduced their workload, and 76% (n=21) recommended continued collaboration with the Wellness Learning Community for further process improvements and program creation.

## Discussion

This project incorporated several key components that improved workflow and enhanced healthcare providers' well-being. By streamlining administrative tasks and minimizing unnecessary burdens, the initiative enabled providers to devote more time and attention to direct patient care-an essential part of their roles. 

One critical focus of the project was fostering interprofessional collaboration. By involving teams from social work, case management, and other key stakeholders, the hospital developed a more efficient and effective workflow. This teamwork-driven approach is vital for improving patient care outcomes, as research consistently shows that collaboration leads to safer and higher-quality care [8]. 

Research also underscores the importance of healthcare providers' physical and mental well-being in reducing medical errors. For example, a 2021 study published in the American Journal of Critical Care found that critical-care nurses in better health, working in organizations prioritizing employee wellness, reported fewer medical errors [9] . Similarly, a 2022 Healthcare Executive article, "The Secret to Safer Patients: Workforce Wellness," emphasized the direct link between workforce wellness and patient safety [10]. This connection was reinforced by the Minimizing Error, Maximizing Outcome study funded by the Agency for Healthcare Research and Quality, which revealed that physician stress and burnout were linked to a higher likelihood of medical errors [11]. 

The redesign of the workflow process for the 3122 form reduced both administrative workload and error risks. These changes allowed hospitalists to focus more on patient care, improving both staff well-being and patient safety [12]. The impact of illegible handwriting on medical errors is well-documented. The Institute of Medicine (IoM) estimated that medical errors cause 44,000 to 98,000 preventable deaths annually in the U.S., with approximately 7,000 attributed to illegible handwriting [13]. Transitioning to electronic prescriptions has been shown to mitigate these errors, improving both safety and efficiency [14]. 

While this project specifically addressed the 3122 form, its principles of workflow optimization and burden reduction can be applied across various healthcare settings. It demonstrates how improving administrative processes can relieve provider workload, enabling a greater focus on patient care [15]. Integrating wellness initiatives into quality improvement efforts enhances both provider well-being and patient safety, fostering a healthier work environment and better outcomes for patients [16]. 

There are some limitations to this initiative. This initiative was conducted at a single community hospital with a relatively small sample size, limiting the generalizability of the findings. The assessment occurred shortly after implementation, so the long-term impact on workload and burnout remains unknown. Additionally, the results were based on self-reported survey responses, which are inherently subjective and may not fully reflect objective changes in workflow or burden. While the intervention aimed to reduce transcription-related medical errors, no direct measurement of patient safety outcomes was conducted.

## Conclusions

This project demonstrates the powerful impact of targeting specific, modifiable workflow inefficiencies as a means of supporting clinician well-being. Rather than relying solely on wellness programs that focus on self-care or resilience, this intervention addressed one of the root causes of burnout: administrative overload. By listening to frontline hospitalists and identifying a concrete, high-friction task, the team was able to develop a practical, sustainable solution that directly improved physicians’ daily experience.

The collaborative nature of this project was central to its success. Engaging social work, case management, and hospital leadership allowed for a more efficient redistribution of tasks, aligning responsibilities with appropriate scopes of practice. This kind of interprofessional teamwork not only lightens the load for individual clinicians but also improves overall system efficiency and safety. Importantly, the positive response from hospitalists following the intervention points to a readiness for more of these changes. Providers want to be part of system improvement; they often just need a framework and a forum in which to participate.
